# Macrovascular complication phenotypes in type 2 diabetic patients

**DOI:** 10.1186/1475-2840-12-20

**Published:** 2013-01-18

**Authors:** Giuseppe Papa, Claudia Degano, Maria P Iurato, Carmelo Licciardello, Raffaella Maiorana, Concetta Finocchiaro

**Affiliations:** 1 Unit of Metabolic and Endocrine Diseases, “Centro Catanese di Medicina e Chirurgia” Clinic, via Battello 48, Catania, 95126, Italy

**Keywords:** Type 2 diabetes mellitus, Macrovascular disease, Cardiovascular risk factors, Metabolic syndrome

## Abstract

**Background:**

Macrovascular diseases (MVD) in type 2 diabetes mellitus (T2DM) are often considered all together, without discriminating the areas involved. The aim of our study was to analyse MVD prevalence in a large population of T2DM patients by dividing the cases into subgroups according to MVD sites (NMVD, no MVD; NSCS, non-significant carotid stenosis; CBVD, cerebrovascular disease; CAD, coronary artery disease; PAD, peripheral artery disease; PVD, polyvascular disease) and studying the anthropometric, clinical and laboratory parameters in each group.

**Methods:**

A diabetic outpatient cohort (n = 1199) was retrospectively studied. Demographic, clinical and laboratory parameters were included in analyses. A thorough cardiovascular history as documented by previous medical records (including medical and hospital records) and vascular laboratory studies (including standardised electrocardiogram, echocardiogram, provocative tests for cardiac ischaemia, ankle/brachial index, duplex ultrasonography of the carotid and lower limbs and, in selected cases, computed tomography angiography, carotid and peripheral arteriography and evaluation of transcutaneous oxygen pressure), was collected for all of the patients. Standardised procedures were used to assess microvascular complications as well as metabolic syndrome (Mets).

**Results:**

The unadjusted MVD prevalence was 46.4% among the participants. The majority of patients with MVD were in the PVD group. In the multivariate analysis, age, male sex and diabetes duration were independent risk factors for PAD and PVD (P < 0.01). A low HDL-C value was an independent risk factor in the CAD and PVD groups (P = 0.03). Very high frequencies of MetS were observed in the PAD and PVD groups (94.9 and 95.7% respectively). The most MetS diagnostic criteria were recorded among members of the CAD group (all or all-1 criteria were present in 73% of patients). The average age in the CAD group (64.5 y) was comparable to that of the NMVD group. Microvascular complications were more frequent in the PAD and PVD patients.

**Conclusion:**

Phenotypic heterogeneity is associated with different macrovascular complications in T2DM patients. These findings might have clinical implications for developing diagnostic and therapeutic strategies targeting type 2 diabetes.

## Background

Cardiovascular complications are the leading cause of morbidity and mortality among patients with type 2 diabetes, and cardiovascular disease (CVD) risk is 2- to 8-fold higher in the diabetic population than it is in non-diabetic individuals of a similar age, sex and ethnicity [1,2]. Furthermore, macrovascular complications are the largest contributor to the direct and indirect costs of diabetes [3]. However, when describing cardiovascular disease in diabetic patients, the patient population is lumped together as a heterogeneous group with CVD, without separation into subgroups according to the macrovascular disease type. Furthermore multiple vascular sites are often simultaneously involved in type 2 diabetic patients, which contributes to the highest cardiovascular risk in this population. To the best of our knowledge, no extensive clinical studies of type 2 diabetic subjects have stratified patients according to their macrovascular complications. The aim of our retrospective study was to evaluate macrovascular disease in a large cohort of type 2 diabetic adults by dividing the subjects into several subgroups according to the vascular areas that were involved. We also studied differences between these subgroups in their anthropometric, clinical and laboratory characteristics, as well as their MetS prevalence/severity.

## Methods

### Subjects

In this observational, cross-sectional survey, we studied 1199 subjects with type 2 diabetes who had been referred to us by general practitioners or other specialists for diabetes management and/or chronic complication assessment between January 1, 2008 and January 1, 2011. The subjects were divided into 6 subgroups: *NMVD*, no macrovascular disease; *NSCS*, non-significant carotid stenosis; *CBVD*, cerebrovascular disease; *CAD*, coronary artery disease; *PAD*, peripheral artery disease; and *PVD*, polyvascular disease. The data were collected from a diagnostic Day-Hospital in our centre and were retrospectively analysed. The exclusion criteria were as follows: acute illnesses, advanced renal disease (creatinine clearance ≤15 ml/min or dialysis), chronic active hepatitis (liver transaminases ≥2 times higher than the normal range and/or positive viral hepatitis B or C serology), or glucocorticoid therapy. The local ethics committee approved the study.

### Clinical and laboratory measurements

Body weight was measured in light clothing without shoes to the nearest half kilogram. Height was measured to the nearest half centimetre. Body mass index (BMI) was calculated as the weight (kg) divided by height^2^ (m). Waist circumference (WC, to the nearest half centimetre) was measured in the standing position at the umbilicus. Arterial blood pressure was taken with a standard mercury blood pressure meter. Three blood pressure readings were obtained at 1 min intervals, and the systolic and diastolic pressure readings were averaged and used for the analysis. Venous blood was drawn in the morning at ward admission after a 10–12 h overnight fast. All of the biochemical parameters were evaluated with standard laboratory procedures. All of the patients were tested for viral hepatitis B and C. LDL cholesterol was calculated by the Friedewald formula except when the serum triglyceride concentration was >400 mg/dL. HbA_1c_ was measured by high-performance liquid chromatography (HPLC); the upper normality limit for the laboratory was 5.9%. A daily glycaemic profile with 6 finger-prick tests (One Touch Ultra, LifeScan, Milpitas, California, USA) was also obtained from all of the patients. Metabolic Syndrome (MetS) was diagnosed using the AHA-NHLBI criteria [4], by the presence of diabetes and ≥2 of the following components: 1) WC >102 cm in men and >88 cm in women; 2) triglycerides >1.7 mmol/L (150 mg/dL) or fibrate/fish oil users; 3) HDL <1.0 mmol/L (40 mg/dL) in men and <1.29 mmol/L (50 mg/dL) in women; and 4) blood pressure ≥130/85 mmHg or receiving blood pressure reduction treatment.

### Macrovascular complication evaluation

A thorough cardiovascular history, as documented by previous medical records (including medical and hospital records) and vascular laboratory studies (including standardised electrocardiogram, echocardiogram, ankle/brachial index, duplex ultrasonography (DUS) of the carotid and lower limbs and provocative tests for cardiac ischaemia), was collected for all of the patients.

#### Non-significant carotid stenosis (NSSC) diagnosis

Asymptomatic patients with 50-69% carotid stenosis in any section as detected by DUS were diagnosed with NSSC.

#### Cerebrovascular disease (CBVD) diagnosis

Patients with a history of TIA, previous stroke (as confirmed by CT/MRI brain scan), or previous significant/symptomatic carotid stenosis (patients had undergone surgical or endoluminal interventions) were diagnosed with CBVD. However, asymptomatic patients with significant stenosis (≥70% in any section as detected by DUS and confirmed by computed tomography angiography (CTA)) were also included in this group. Patients who were undergoing interventional procedures were sent directly for a carotid arteriography.

#### Coronary artery disease (CAD) diagnosis

We included in this group all of the patients with previous acute coronary sindromes (ACS) diagnoses (STEMI, NSTEMI, uns4angina) whether or not they had endoluminal or surgical revascularisation procedures. We also included those patients with ischaemic heart disease and stable angina. All of the patients were subjected to cardiac examination, ECG and echocardiogram to confirm the previous diagnosis. Patients with ECG abnormalities or symptoms that were suggestive of ischaemia and/or with provocative tests suspected for myocardial ischaemia were sent for a coronary angiography to confirm the diagnosis or endoluminal treatment.

#### Peripheral artery disease (PAD) diagnosis

Patients who had endoluminal or surgical revascularisation interventional procedures, had previous amputations, or had been diagnosed with ischaemic ulcers were diagnosed with PAD. All of the symptomatic patients (Fontaine’s classification stage II-IV) who were confirmed by ABI and DUS were diagnosed with PAD. Asymptomatic patients with an ABI <0.9 who had significant stenosis or occlusions as determined by DUS (and confirmed by peripheral CTA) were also included in the PAD group. Patients with suspected critical limb ischaemia were evaluated by transcutaneuos oxygen pressure (TcPO2); if the results were <30 mmHg, then the patients were referred to angiography to perform endoluminal procedures.

#### Polyvascular disease (PVD) diagnosis

When two or more of the above conditions were present at the same time, the patients were diagnosed with PVD.

#### No macrovascular disease (NMVD) diagnosis

Patients who did not meet the criteria above were not considered to have cardiovascular disease.

### Microvascular complication evaluations

Microvascular complications were evaluated using fundus oculi and/or fluorescence angiography to assess retinopathy, urinary albumin excretion and eGFR calculation to assess nephropathy, and the 10g monofilament test and vibration perception threshold analysis to assess peripheral neuropathy. Microalbuminuria was defined as urinary albumin excretion between 30 and 299 mg/day on at least 2 of 3 occasions. The eGFR was calculated using the MDRD formula [estimated GFR (mL/m/1.73 m^2^) = 186 × creatinine (mg/dl)^-1.154^ × age (yy)^-0.203^ × 0.742 (if female) × 1.210 (if of black ethnicity)] [5]. Diabetic retinopathy was defined as any diabetes-linked retinal injury. Diabetic peripheral neuropathy was diagnosed based on neuropathic symptoms, insensitivity to a 10g monofilament and an abnormal vibration perception threshold.

### Statistical analysis

The data were tested for normalcy using the Kolmogorov-Smirnov test, which indicated a non-Gaussian distribution for all continuous variables; therefore, the results were reported as a median value and an interquartile range (IQR, 25^th^-75^th^ quartiles). Univariate analysis was performed with the non-parametric Kruskal-Wallis test and Dunn’s multiple comparison post-hoc test to compare each of the continuous variables of interest among the macrovascular groups. The qualitative variable data were expressed as frequencies, and the groups were compared using the chi-square test. Multivariate logistic regression analysis was used to determine whether traditional cardiovascular risk factors (sex, age, smoking status, diabetes duration, WC, SBP, DBP, LDL-C, HDL-C, triglyceride levels, HbA1c, FPG, use of antihypertensive and lipid-lowering drugs and the presence of AHA/NHLBI-defined metabolic syndrome) were associated with prevalent CVD (dependent variable, MVD; reference group, NMVD), and the results were expressed as odds ratios (ORs) ± 95% CI. The ORs were calculated using exponential logistic regression coefficients. A p-value of less than 0.05 was considered to be statistically significant. All of the analyses were performed with SPSS version 17 (Chicago, IL, USA) and Prism software (GraphPad, USA).

## Results

**Figure 1:**
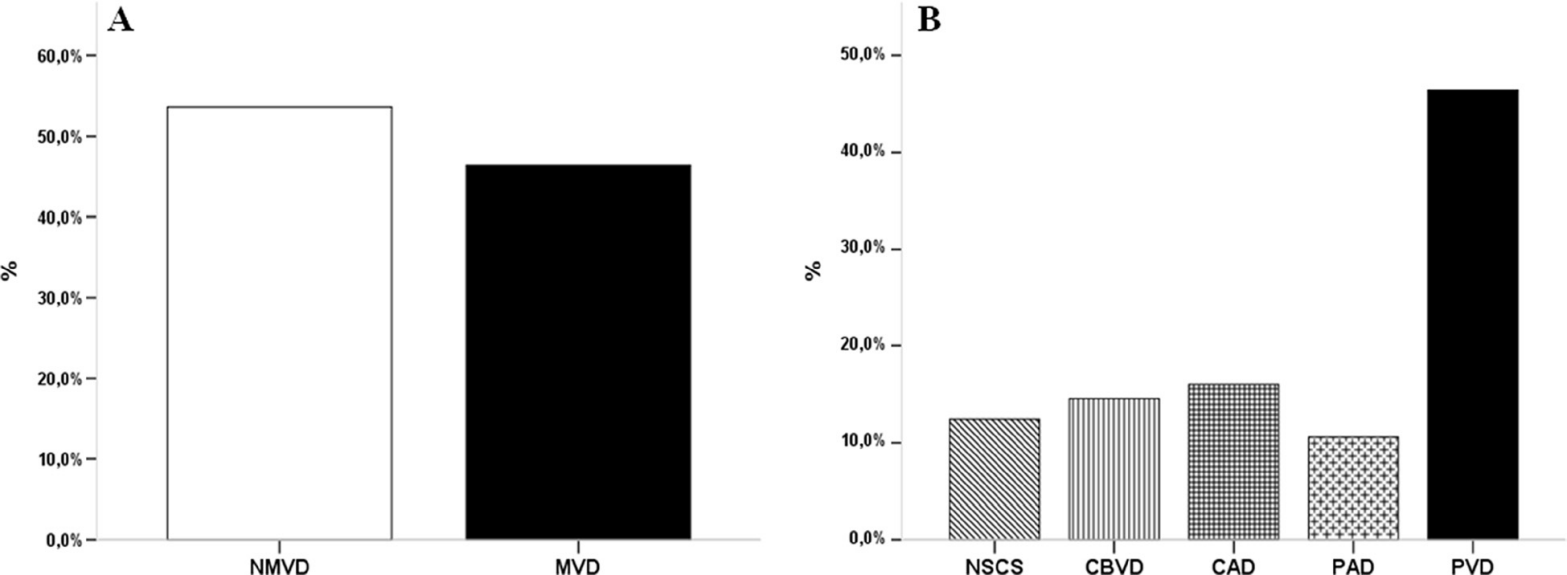
**Panel A**: **Macrovascular complication prevalence in our population. Panel B: Macrovascular subgroup prevalence in patients with macrovascular complications.** The data are frequencies.

Clinical and biochemical patient characteristics are presented in Table 1. In total, 643 (53.6%) patients had no evidence of macrovascular disease, and the remaining 556 (46.4%) had MVD (see Figure 1, panel A). There were 12.4% of the MVD patients who were classified as NSCS, 14.6% as CBVD, 16% as CAD, 10.6% as PAD, and 46.4% as PVD (Figure 1, panel B).

**Table 1:** **Clinical and metabolic patient variables** (**all of the patients and grouped according to macro**-**vascular involvement**)

**Variables**	**All**	**NMVD** (**I**)	**NSCS** (**II**)	**CBVD** (**III**)	**CAD** (**IV**)	**PAD** (**V**)	**PVD** (**VI**)	***p differences*among the groups**^**a**^
N patients (%)	1199	643 (53.6)	69 (5.7)	81 (6.8)	89 (7.4)	59 (4.9)	258 (21.5)	_
Age (yy)	67 (58–75)	63 (55–72)	66 (59–71)	73 (66–79)	65 (55–75)	72 (65–76)	73 (67–78)	I≠III,V,VI***, II≠III**,VI*** III≠IV***, IV≠V*,VI***
Sex %M	49.5	45.3	39.1	37	58.4	61	61.2	***
DM2 duration (yy)	12 (6–20)	10 (5–18)	10 (5–15.5)	12.5 (7–20)	14 (6–20)	20 (10–30)	20 (10–28.3)	I≠V,VI***, II≠V,VI***, III≠VI*, IV≠VI**
Weight (Kg)	80 (69.5-92.7)	81 (71.1-94.4)	80.1 (67.3-93.8)	74.6 (65.1-85.5)	85.7 (74.4-98.6)	75.2 (67–90)	76.5 (65.4-87.8)	I≠III,VI**, III≠IV***, IV≠VI**
BMI (Kg/m^2^)	31.1 (27.6-35.5)	31.5 (28.1-36.4)	31.5 (26.5-36.6)	29.7 (27.1-33.6)	32.2 (27.6-37)	30.3 (26.3-34.9)	30 (26.3-33.8)	I≠VI**, IV≠VI*
WC (cm)	105 (97–118)	106 (97–120)	105.5 (94.3-119)	102 (96–110.5)	110 (98–120)	103 (98–115.8)	104 (97–116)	ns
Smoking history %	27.3	26.9	20.3	14.8	25.8	33.9	32.6	***
SBP (mmHg)	130 (120–140)	130 (120–140)	130 (120–140)	130 (120–140)	130 (120–140)	130 (120–145)	130 (120–140)	ns
DBP (mmHg)	80 (70–80)	80 (70–80)	80 (70–80)	70 (70–80)	80 (70–80)	70 (70–80)	80 (70–80)	ns
TC (mg/dl)	178 (151–210)	183 (157–212)	201 (175–221)	176 (153–204)	162 (132–193)	179 (156–212)	165 (135–196)	I≠IV,VI***, II≠IV,VI***
HDL-C (mg/dl)	45 (38–55)	47 (39–57)	49 (39–58)	47 (41–60)	42 (35–50)	45 (39–55)	43 (35–52)	I≠IV*,I≠VI***, III≠IV,VI*
LDL-C (mg/dl)	102(77–131)	105(83–133)	121(99–144)	101(76–129)	82 (64–116)	102(74–130)	91 (70–123)	I≠IV,VI***, II≠IV,VI***
TG (mg/dl)	124 (91–166)	123 (91–161)	125 (96–173)	117 (82–149)	126 (94–184)	123 (90–170)	130 (91–176)	ns
HbA1c (%)	8.1 (7–9.5)	8.1 (7.1-9.7)	8 (6.9-10.2)	8.2 (6.8-9.5)	8.1 (7.2-9.5)	7.7 (6.8-9)	8 (7–9.3)	ns
FPG (mg/dl)	174 (139–224)	174 (138–230)	177 (148–232)	169 (138–213)	182 (142–222)	177 (140–218)	174 (137–219)	ns
2h-BG (mg/dl)	201 (160–265)	203 (160–270)	219 (174–286)	195 (160–232)	205 (160–270)	200 (167–239)	200 (155–254)	ns
∆BG (prandial)	34 (−6-76)	35 (−3-79)	44 (14–81)	39 (−8-88)	38 (−1-61)	31 (−22-58)	22 (−16-69)	ns
MetS %	88	83.1	91.3	88.9	89	94.9	95.7	***
3-4 MetS factors %	61	55.7	62.3	64.2	73	69.5	66.7	***
eGFR	84 (65–101)	89 (74–106)	87 (61–100)	79 (65–99)	82 (57–103)	66 (49–88)	68 (50–88)	I≠III*,V,VI***, II≠V*, II≠VI**, IV≠VI*
Albuminuria %	25.9	24.3	21.9	25.3	31.8	33.3	27.2	ns
DR %	37.3	28.6	33.3	40.7	38.2	78	49.2	***
DN %	49.7	39.0	42.0	53.1	43.8	88.1	70.5	***
Statin users %	65	55.4	69.6	70.4	76.4	81.4	78.7	***
AH user %	80.4	72.3	79.7	85.2	91	96.6	91.9	***
OHA users %	38.3	43.2	49.3	42	39.3	20.3	25.6	***
OHA+BI users %	13.6	16.5	11.6	8.6	10.1	10.1	10.5	***
MDI users %	48.1	48.1	39.1	49.4	50.6	69.3	63.9	***

Data are medians (IQR, 25^th^–75^th^ quartiles) or proportions; ^a^Kruskal-Wallis one-way ANOVA with Dunn’s multiple comparison post-hoc test and *χ*^2^-test; ns, not significant; *p<0.05, **p≤0.01, *** p≤0.001; 2h-BG, 2h glycaemia after meals; ∆ BG, ∆ post-preprandial glycaemia; DR, Diabetic retinopathy; DN, Diabetic Neuropathy; eGFR was expressed as mL/min/1.73 m^2^.

### Univariate analyses

#### Macrovascular disease, anthropometric and clinical data

Patients with CBVD, PAD and PVD were older than were patients in the other groups. CAD patients were similar in age to those without complications and to those in the NSCS group. Male patients were more numerous in CAD, PAD and PVD groups, whereas female patients were more numerous in the other groups.

Diabetes duration was significantly greater in the PAD and PVD patients. BMI was significantly lower in the PVD group compared to the NMVD and CAD groups.

#### Macrovascular disease and metabolic parameters

We found no differences in the metabolic parameters among the 6 subgroups. The HbA1c levels, FPG levels, post-prandial glucose levels, and Δpre-postprandial plasma glucose levels were comparable among all of the groups. All of the data were outside of the normal ranges because the patients had poor glycaemic control at this first observation in our centre.

#### Macrovascular disease and lipoprotein profile

The extensive use of statins across all of the groups strongly influenced the lipid profile in our patients. The total cholesterol levels were higher in patients without complications or with NSCS compared with CAD or PVD patients, and the LDL-C values followed the same trend. HDL-C was significantly lower in the CAD and PVD patients compared to those in the NMVD and CBVD groups. No significant differences in the triglyceride values were found among the groups.

#### Macrovascular disease and MetS

**Figure 2:**
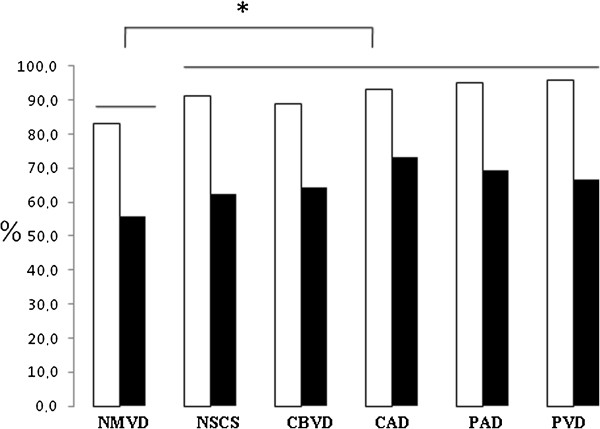
**MetS prevalence (white columns) and** “**severity**” **(black columns) (presence of 3 or 4 AHA/NHLBI classification criteria) in the subgroups.** The data are frequencies. *, significant at p ≤ 0.001 from Chi-Square test.

As expected, metabolic syndrome prevalence was significantly greater in patients who belonged to the macrovascular groups than in patients who did not have complications. The highest prevalence was recorded in the PAD (94.9%) and PVD (95.7%) patients. The simultaneous presence of 3 or 4 diagnostic MetS criteria (indicating MetS severity) was more frequent in CAD patients (73%) (Figure 2).

#### Macrovascular disease and microvascular complications

Microvascular complications (diabetic retinopathy, diabetic nephropathy and diabetic neuropathy) were significantly more prevalent in PAD and PVD subjects than in the other subjects.

### Multivariate Analysis

Multivariate logistic regression analysis was used to determine whether traditional cardiovascular risk factors (sex, age, smoking status, diabetes duration, WC, SBP, DBP, LDL-C levels, HDL-C levels, triglyceride levels, HbA1c levels, FPG levels, use of antihypertensive and lipid-lowering drugs and the presence of AHA/NHLBI-defined metabolic syndrome) were associated with prevalent CVD (dependent variable, MVD; reference group, NMVD),and the data were expressed as odds ratios (ORs) (see Table 2). In this analysis, in all of the macrovascular groups except for the CAD and NSCS groups, age was significantly associated with macrovascular disease. Male sex and diabetes duration were significantly associated only in the PAD and PVD groups. Reduced HDL-C was independently associated in the CAD and PVD groups. Statin utilisation was independently associated in all of the macrovascular groups with the exception of the NCSC group. MetS diagnosis was independently associated with only the PVD group. In the logistic regression model, the inclusion of logarithmically transformed values of HDL-C, LDL-C and triglycerides did not change the results. Similarly, when excluding MetS, logistic model coefficients were almost unchanged (data not shown).

**Table 2:** Multivariable logistic regression analyses

	**NSCS**			**CBVD**			**CAD**			**PAD**			**PVD**		
	**OR**	**95**% **CI**	**p**	**OR**	**95**% **CI**	**p**	**OR**	**95**% **CI**	**p**	**OR**	**95**% **CI**	**p**	**OR**	**95**% **CI**	**p**
Gender (male)	1.03	0.54–1.96	0.94	1.35	0.70–2.61	0.37	1.53	0.85–2.74	0.16	2.90	1.37–6.12	**0**.**005**	2.39	1.53–3.73	**0**.**0001**
Age	1.03	1.0–1.06	0.09	1.10	1.06–1.14	<**0**.**0001**	1.02	0.99–1.04	0.28	1.09	1.05–1.14	<**0**.**0001**	1.11	1.08–1.14	<**0**.**0001**
WC	0.99	0.97–1.01	0.36	0.97	0.95–0.99	**0**.**01**	0.99	0.98–1.01	0.45	0.98	0.96–1.00	0.07	0.99	0.97–1.00	**0**.**04**
Diabetes duration	0.98	0.94–1.01	0.17	0.99	0.96–1.02	0.68	1.01	0.98–1.04	0.60	1.05	1.02–1.08	**0**.**004**	1.04	1.02–1.06	**0**.**0001**
Smoking History (yes)	1.81	0.52–6.38	0.35	1.44	0.35–5.9	0.62	0.70	0.26–1–90	0.49	2.02	0.63–6.43	0.24	0.85	0.42–1.73	0.65
SBP	1.02	0.99–1.04	0.18	1.01	0.98–1.03	0.46	0.97	0.94–0.99	**0**.**007**	1.03	1.01–1.06	**0**.**01**	0.99	0.98–1.01	0.73
DBP	0.98	0.94–1.02	0.41	0.99	0.95–1.04	0.84	1.05	1.01–1.10	**0**.**009**	0.95	0.91–0.99	**0**.**03**	1.01	0.98–1.04	0.75
AH user (yes)	0.96	0.45–2.02	0.91	1.25	0.53–2.94	0.61	2.93	1.19–7.23	**0**.**02**	8.4	1.02–68.4	**0**.**04**	1,55	0.83–2.89	0.17
HDL-C	0.99	0.97–1.02	0.66	0.99	0.97–1.02	0.74	0.97	0.95–0.99	**0**.**03**	0.97	0.95–1.00	0.06	0.98	0.97–0.99	**0**.**03**
LDL-C	1.00	0.99–1.01	0.16	1.00	0.99–1.01	0.41	0.99	0.98–0.99	**0**.**02**	1.00	0.99–1.01	0.66	1.00	0.99–1.00	0.09
Triglycerides	1.00	0.99–1.00	0.45	0.99	0.99–1.00	0.18	1.00	0.99–1.00	0.70	1.00	0.99–1.01	0.65	1.00	0.99–1.00	0.48
Statin users (yes)	1.51	0.80–2.85	0.21	4.11	1.94–8.60	**0**.**0002**	2.31	1.24–4.30	**0**.**009**	4.92	1.99–12.10	**0**.**0006**	3.42	2.13–5.47	<**0**.**0001**
HbA1c	0.94	0.76–1.15	0.53	1.08	0.87–1.34	0.48	0.93	0.76–1.13	0.45	0.78	0.60–1.03	0.09	0.98	0.84–1.13	0.75
FPG	1.00	0.99–1.01	0.26	0.99	0.99–1.00	0.35	1.00	0.99–1.01	0.33	1.00	1.00–1.01	0.51	1.00	0.99–1.00	0.95
MetS (yes)	1.29	0.47–3.57	0.62	1.33	0.43–4.12	0.62	1.10	0.37–3.27	0.86	1.81	0.34–9.73	0.49	2.30	1.01–5.29	**0**.**04**

Relationship (ORs) among selected variables and the macro-vascular groups (Dependent variable, macrovascular groups; reference group, NMCV). Values in bold are significant.

## Discussion

The first goal of our study was to assess the prevalence of different macroangiopathic conditions in our patients. Our study demonstrates that 46.4% of our patient population has macrovascular disease. It is well known that CVD prevalence, incidence, and mortality are strikingly greater in the diabetic than in the non-diabetic population. According to the World Health Organization, CVD prevalence in diabetic patients ranges from 26 to 36% [6]. The significantly higher prevalence of macrovascular complications in our patients is likely linked to patient characteristics (average age, diabetes duration, poor glycaemic control over time) and due to our inclusion of NSCS patients. As expected, almost half of our patients had polydistrectual involvement.

Advanced atherosclerotic vascular changes are often preceded by impairment of endothelium-dependent vasodilation, vascular smooth muscle dysfunction and increased arterial stiffness. Today all of these factors are recognized as predictors of vascular dysfunction in T2DM patients [7]. Atherosclerotic disease usually causes systemic involvement, which is more frequent in the diabetic population [8]. Moreover, atherosclerosis in diabetic patients is different from that in non-diabetic subjects because both pathologic studies and angiographic reports in individuals with coronary heart disease and PAD have shown that diabetic patients have more blood vessels involved, with a more diffuse atherosclerotic lesion distribution [9,10].

Genetic studies have also shown that different macrovascular phenotypes (as CAD and CBVD) and T2DM share a major linkage at the chromosome 12q24 locus. In this regard, the gene of proteasome modulator 9 (PSMD9) is linked to macrovascular pathology of T2DM [11].

Estimated twenty years incidence of different cardiovascular complication phenotypes (ischaemic heart, myocardial infarction, heart failure, cerebrovascular disease, amputation of lower limbs) was evaluated in a Mexican diabetic population by a simulation model indicating that a large portion of this diabetic population is at risk of myocardial infarction and cerebrovascolar disease in subsequent years [12]. Also in our study, among patients with macrovascular-isolated events, the majority of patients were placed in the CAD (16%) and CBVD (14.6%) groups.

PAD, CAD and CBVD risk factors are similar and are also typical atherosclerosis risk factors. These risk factors include smoking, dyslipidaemia, diabetes and hypertension. However, specific risk factors, such as genetic background, could be more important for the development of macro-vascular disease at certain sites. To the best of our knowledge, no study has compared the anthropometric, clinical and laboratory features in type 2 diabetic patients by stratifying patients according to the type of macrovascular involvement. For this reason, as a second study target, we tested the hypothesis that different macrovascular involvement types might correspond to different phenotypes. By analysing the anthropometric and clinical characteristics of subgroups of patients, we found that PAD and PVD patients were older, had had diabetes for a longer period of time, were more likely to have been smokers and had a lower weight, BMI and WC than did the patients in the other groups. A multivariate analysis demonstrated that age, male sex, diabetes duration and the use of statins were the most important independent PAD and PVD risk factors. It is noteworthy that the same characteristics were not found to be independent risk factors in the CAD group and that the average patient age in this group was comparable to that of the population without macrovascular complications. The question arises of whether a genetically determined predisposition alone may explain the early onset of cardiac involvement in the CAD group. In recent years, many genetic risk factors for both diabetes and coronary artery disease have been discovered through genome-wide association studies. Genetic aspects of diabetes, diabetic macrovascular complications and CAD may share mechanisms, leading to a common effector hypothesis. However, only a few genetic risk factors could be identified that modulate the risk for both conditions. Polymorphisms in the TCF7L2 and near the CDKNZA/B genes may be of great importance for CAD development because these genes modulate both conditions and are not necessarily related to hyperinsulinaemia or hyperglycaemia [13].

The complex interactions between genetic and environmental factors on cardiometabolic risk were analyzed by twin studies indicating that some cardiometabolic risk factors had strong heritability (as weight, waist circumference, SBP, DBP) while others were substantially influenced by environmental factors [14].

By analysing glico-metabolic parameters (HbA1c, fasting glucose, postprandial glucose and Δ post-preprandial blood glucose) we found no differences among the six subgroups. Despite a clear association between diabetes and atherosclerotic vascular disease, the underlying mechanism responsible for the two diseases is not fully understood. The relative importance of “non-glycaemic” risk factors and hyperglycemia “per sè” has always been debated [15]. Results on the causal relationship between hyperglycemia and macroangiopathy have been contradictory. Plenty of evidence suggest a significative relationship between HbA1c levels, post-prandial hyperglycemia, and risk of CV events and adverse outcomes especially in overweight and obese patients [16,17] . However, recently three major studies, ACCORD [18], ADVANCE [19] and VADT [20] evaluated the impact of attaining euglycemia (ACCORD) or near-euglycemia (ADVANCE and VADT) in patients with long-lasting diabetes and high cardio-vascular risk. None of these studies, either individually or on pooled analysis, demonstrated any reduction in all cause or cardiovascular mortality, although a meta-analysis revealed a 15-17% reduction in the incidence of non-fatal myocardial infarction in those patients exposed to tight glucose control [21]. A higher mortality was observed in the intensive glucose control arm of ACCORD, leading to the premature termination of the glucose-lowering component of this study. The weak association between glycemic control and macro-vascular disease observed in UKPDS [22] has been confirmed and amplified by these recent intervention studies. Accordingly, our data show that glycated hemoglobin, post-prandial and fasting glucose were similar in each subgroup considered. However, the relationship between hyperglycemia and macrovascular complications is made even more complex by the potential role of epigenetic mechanisms, as the metabolic memory, by which a prior exposure to hyperglycemia predisposes diabetic patients to the continuing development of vascular diseases despite a subsequent good glycemic control [23]. Furthermore, in our study, referral bias (diabetic patients with poor metabolic control referred to us for the first time) did not allow to clarify the causal or temporal relationship among macrovascular events and glycemic control over time.

By analysing the lipid profile, we found a clear difference in HDL-C among the groups, which was lower in CAD, PAD, and PVD patients. These data are in accordance with other scientific evidence that support the importance of this macroangiopathy-associated risk factor. In a multivariate analysis, we also confirmed that reduced HDL-C values are independent CAD and PVD risk factors (the association was borderline significant for the PAD group). The importance of HDL-C as “target therapy” is now emerging from recent cardiovascular trials of CETP (cholesteryl ester transfer protein) inhibitors, such as Anacetrapib [24] and Evacetrapib [25]. This new pharmacological group is very promising for cardiovascular risk reduction either when administered alone or in conjunction with conventional therapy.

Many reports have suggested that metabolic syndrome may precede/predict vascular disease. It has been reported that insulin resistance and metabolic syndrome increase the risk of new cardiovascular events also in patients without known diabetes but with manifest arterial disease [26]. In type 2 diabetes, metabolic syndrome is highly prevalent and often precedes hyperglycaemia onset [27,28]. Furthermore, insulin resistance and metabolic syndrome predict atherosclerosis in type 2 diabetic patients [29]. Our findings support the clinical relevance of MetS component detection, which may be a simple, quick tool to stratify diabetic patients according to the expected macrovascular complication severity (as a polydistrectual disease).

Microvascular complications were not equally present in the various groups. We found more diabetic nephropathy, as assessed by micro/macroalbuminuria or by calculating the eGFR in the CAD, PAD and PVD groups. Diabetic neuropathy was more frequent in the PAD and PVD groups, and diabetic retinopathy was strikingly present in the PAD group. As expected, patients in the CAD, PAD and PVD groups were the largest statin and antihypertensive drug users, and those in the PAD and PVD groups were more frequently treated with multiple insulin injections.

### Strengths and limitations

Macrovascular complication phenotyping in type 2 diabetic patients has not yet been reported. Such phenotyping may have diagnostic and therapeutic implications in type 2 diabetes management. An additional strength of our study is the large number of patients who were studied in a single clinical centre, with each diagnostic examination being performed by a single operator. However, we recognise that there are some limitations to our study. First, the retrospective cross-sectional design precluded the establishment of causal or temporal relations among macrovascular events and other features in our diabetic population. In addition, this study mainly included older diabetic subjects who had unsatisfactory glycaemic control, who may not have been representative of the general diabetic population.

## Conclusion

Our study shows that nearly half of diabetic patients, especially those who are elderly and male with a long disease duration, have polydistrectual atherosclerotic involvement.

The characterization of different phenotypes may have a clinical significance for the everyday clinician.

Firstly, different factors were associated with isolated coronary artery disease, like a mean age comparable to that of patients without macro-vascular complications and a high frequency of severe metabolic syndrome. These data raise the interesting hypothesis that coronary artery disease, metabolic syndrome and diabetes may be genetically linked in some individuals. Further studies may clarify whether this is because of genetic background and/or because coronary heart disease might be the first event in polydistrectual atherosclerotic involvement.

Secondly, low HDL-C values are a marker of PAD, CAD and PVD, confirming the importance of HDL-C as a potential therapeutic target. Alternatively, lifestyle modification strategies to prevent a low HDL-cholesterol should be implemented.

## Abbreviations

ABI: Ankle-brachial index; ACS: Acute coronary syndrome; BMI: Body mass index; CAD: Coronary artery disease; CAS: Carotid artery stenting; CBVD: Cerebrovascular disease; CEA: Carotid endarterectomy; CLI: Critical limb ischaemia; CT: Computed tomography; CTA: Computed tomography angiography; CVD: Cardiovascular disease; DBP: Diastolic blood pressure; DUS: Duplex ultrasonography; eGFR: Estimated glomerular filtration rate; FPG: Fasting plasma glucose; MVD: Macrovascular disease; MDIs: Multiple daily injections; MetS: Metabolic syndrome; NMVD: No macrovascular disease; NSCS: Non-significant carotid stenosis; PAD: Peripheral artery diseases; PTA: Percutaneous transluminal angioplasty; PVD: Polyvascular disease; SBP: Systolic blood pressure; TcPO2: Transcutaneuos oxygen pressure; T2DM: Type 2 diabetes mellitus; TIA: Transient ischaemic attack; WC: Waist circumference.

## Competing interests

None of the authors has any conflict of interest.

## Authors’ contributions

GP participated in the overall design, data collection, statistical analysis, data interpretation, writing and critical review of the manuscript. CD, MPI, CL, RM participated in data collection, data interpretation and critically revised the manuscript before final approval. CF supervised the design and conduction of the study, participated in data interpretation and critically revised the manuscript before final approval. All authors read and approved the final manuscript.
